# Beyond barriers: when neurons act immune and immunity acts neural

**DOI:** 10.1038/s41392-025-02494-3

**Published:** 2025-12-02

**Authors:** Sang Wha Kim, Seung Hyeok Seok

**Affiliations:** 1 College of Veterinary Medicine & Institute of Veterinary Science, Kangwon National University, Chuncheon, Gangwon Republic of Korea; 2 Macrophage Lab, Department of Microbiology and Immunology, Seoul National University College of Medicine, Seoul, Republic of Korea; 3 Institute of Endemic Diseases, Seoul National University Medical Research Center (SNUMRC), Seoul, Republic of Korea; 4 Department of Biomedical Sciences, Seoul National University College of Medicine, Seoul, Republic of Korea; 5 Cancer Research Institute, Seoul National University, Seoul, Republic of Korea

**Keywords:** Innate immunity, Neuroimmunology

In a recent study published in *Nature*, Yu et al.^1^ revealed that microglia regulate GABAergic neurogenesis in the prenatal human brain through insulin-like growth factor 1 (IGF1), redefining how the nervous and immune systems intersect during early development.^1^ By demonstrating that microglia-derived IGF1 acts as a trophic cue that promotes the differentiation and integration of inhibitory neurons, this study positions microglia as developmental architects rather than mere immune sentinels. These findings not only illuminate a previously unrecognized neuroimmune mechanism shaping human brain circuitry but also provide a conceptual link between immune signaling and neural circuit assembly, offering potential insights into neurodevelopmental disorders such as autism or epilepsy.

This discovery exemplifies the emerging neuroimmune connectome field conceptualized by Wheeler et al.^2^, who proposed bidirectional neuro-immune regulation as a system-level network across tissues.^2^ The concepts of the “neuroimmune synapse” and “neuroimmune connectome” together describe the structural and functional interfaces through which neurons and immune cells communicate. Specifically, the neuroimmune synapse represents the fundamental cellular unit of this communication – a specialized, polarized cell-to-cell contact with spatially organized expression of signaling molecules and their corresponding receptors. Meanwhile, Wheeler et al.^2^ define the neuroimmune connectome as the totality of neuroimmune interactions forming a system-level network integrating synaptic units across cells, tissues, and organs.^2^

**Fig. 1:**
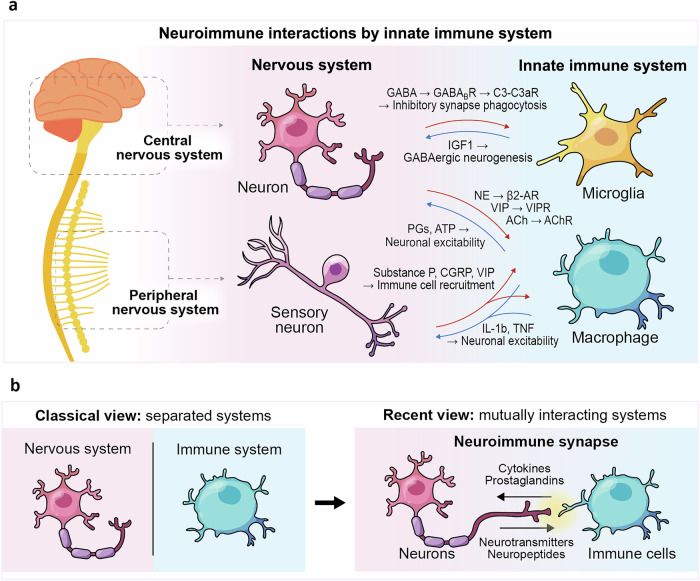
Bidirectional convergence of the nervous and innate immune systems. **a** Representative neuroimmune interactions mediated by innate immune cells across central and peripheral circuits. In the central nervous system, microglial IGF1 signaling drives inhibitory neurogenesis in the prenatal human brain, as demonstrated by Yu et al.^1^, representing a key developmental axis within the neuroimmune connectome. Microglia also regulate inhibitory circuitry by removing inhibitory synapses through GABA_B_ receptor–C3–C3aR–dependent phagocytosis. In the periphery, enteric and sensory neurons engage macrophages via neurotransmitters (NE, VIP, ACh) to shape immune tone, while macrophage-derived prostaglandins (PGs) and ATP modulate neuronal excitability. Sensory neurons also release neuropeptides (Substance P, CGRP, VIP) to recruit immune cells, whereas cytokines (IL-1β, TNF) reciprocally affect neuronal activity. Together, these bidirectional exchanges constitute a neuroimmune connectome linking neural and innate immune systems across tissues. Red arrows indicate the modulation of immune cell states (e.g., recruitment and activation) by neuronal inputs, whereas blue arrows represent neurogenesis and neuronal excitation driven by factors secreted from innate immune cells. **b** Historically, the nervous and immune systems were viewed as independent hierarchies. Recent evidence reveals their continuous communication through neuroimmune synapses, where neurons and innate immune cells exchange cytokines, prostaglandins, complements, neurotransmitters, neuropeptides, and growth factors to coordinate development, homeostasis, and inflammation. ACh acetylcholine, AChR acetylcholine receptor, ATP adenosine triphosphate, β2-AR, β2-adrenergic receptor, C3 complement component 3, C3aR complement C3a receptor, CGRP calcitonin gene-related peptide, ENS enteric nervous system, GABA gamma-aminobutyric acid, GABA_B_R gamma-aminobutyric acid B receptor, IGF1 insulin-like growth factor 1, IL-1β interleukin-1 beta, NE norepinephrine, PGs prostaglandins, TNF tumor necrosis factor, VIP vasoactive intestinal peptide, VIPR vasoactive intestinal peptide receptor

Earlier studies examined this interface in the context of adaptive immunity, emphasizing the interactions between T cells and neurons, yet this framework captured only limited aspects of neuroimmune communication. Emerging evidence shows that innate immune cells directly communicate with neurons (Fig. 1). As another example of innate immune cell interaction with the neural system, in peripheral tissues, neural inputs tune innate immune behavior, with tissue-resident macrophages serving as the principal responders to neuronal cues. This crosstalk is particularly prominent in the gut, where the enteric nervous system directly modulates intestinal macrophages to match the immune tone with physiological needs. Gabanyi et al.^3^ demonstrated that catecholaminergic neurons release neurotransmitters that activate β₂-adrenergic receptors on intestinal macrophages, inducing a tissue-protective, tolerogenic transcriptional program marked by *Arg1*, *Retnla*, and *Ym1*.^3^ These neurogenic signals define macrophage phenotypes as pro-inflammatory during infection and tolerogenic during homeostasis, thereby shaping the mucosal immune landscape.

Neuronal reprogramming of macrophages represents a paradigm shift in innate immunity. Rather than passively responding to cytokines, macrophages dynamically react to neurotransmitters, such as acetylcholine, norepinephrine, and vasoactive intestinal peptide (VIP). In turn, they influence neuronal excitability via prostaglandins and ATP, thereby forming a local regulatory circuit at the tissue-level. Such mutual regulation not only maintains gut homeostasis but also illustrates how the neuroimmune synapse serves as a bidirectional control unit for innate immunity.

If intestinal macrophages demonstrate peripheral reprogramming, microglia epitomize the central nervous system counterpart as an innate immune cell lineage that shapes and remodels neural circuits. Beyond establishing microglia as synapse builders coordinate circuit assembly (Yu et al.^1^), disease states reveal their opposite role as synapse sculptors.^1^ For example, Chen et al.^4^ reported that GABA-dependent, microglia-mediated elimination of inhibitory synapses underlies neuronal hyperexcitability in epilepsy.^4^ Hyperactive GABAergic neurons activate microglial GABA_B_ receptors, triggering complement C3–C3aR signaling and the selective phagocytosis of inhibitory synapses; this targeted removal disrupts the excitatory-inhibitory balance and promotes seizures, which can be reversed by blocking GABA signaling or complement.

Building upon the developmental insights from Yu et al.^1^, recent studies demonstrate that microglia are dual regulators of neural circuitry-fostering synapse formation during development while pruning connections under pathological stress.^1^ This duality highlights the dynamic reciprocity of the neuroimmune synapse, where neural activity guides microglial behavior, and in turn, microglial signaling actively rewires the brain connectome.

The final step in this continuum of convergence lies in recognizing that neurons function as innate immune sensors. In their comprehensive review, Deng et al.^5^ described sensory neurons as integrated components of innate immunity.^5^ Sensory neurons express pattern-recognition receptors (PRRs), including nucleotide-binding oligomerization domain (NOD)-like receptors, toll-like receptors (TLRs), and stimulator of interferon genes (STING) pathway elements, enabling them to directly detect microbial products and damage-associated molecular patterns. Upon activation, neurons release neuropeptides (e.g., substance P, calcitonin gene-related peptide, and VIP) that orchestrate immune cell recruitment and vascular responses. Conversely, cytokines such as IL-1β and TNF modulate neuronal excitability and pain perception, completing a bidirectional feedback loop.

The recognition of neurons as immune effectors fundamentally alters the neuroimmunological landscape. Our understanding of neurons is no longer limited to their capacity to solely transmit electrical information; they also actively interpret and propagate immune signals. When functioning as innate immune sensors, neurons may at last be capable of establishing forms of long-term “neuronal immune memory,” supported by epigenetic evidence. Such possibilities invite a fundamentally new perspective on neurons and mark the evolution of the neuroimmune synapse from being a structural contact to a functional immune organelle – a site where synaptic, metabolic, and immune languages overlap.

At the system level, these cellular interactions integrate into a broader neuroimmune connectome, a network-scale framework mapping the reciprocal communication between neural and immune circuits.^2^ The connectome model unifies scattered observations into a single paradigm of bidirectional convergence, also encompassing other important scenes in which the nervous and immune systems interact across diverse physiological and pathological contexts—such as neuroimmune crosstalk driving cancer progression, pain, and immunotherapy resistance, or modulation of immune responses by sensory neurons in the skin and lungs. Collectively, these examples highlight that the bidirectional convergence paradigm can be observed across virtually all microenvironments where the nervous and immune systems operate in concert.

Collectively, the findings from Yu et al.^1^ —highlighting IGF1-driven microglial regulation of neurogenesis—exemplify how the neuroimmune connectome framework proposed by Wheeler et al.^2^ manifests in human brain development, demonstrating that the nervous and innate immune systems are more deeply entwined than previously recognized.^1,2^ This relationship is not unidirectional but rather reciprocal and integrative: neurons reprogram macrophage immunity, influence microglial state, and erase synapses, and neurons themselves execute immune sensing. The neuroimmune synapse is, therefore, not a passive interface but an active, plastic nexus where two evolutionary programs coalesce.

This understanding opens therapeutic avenues—specifically, by inhibiting complement C3–C3aR–dependent microglial pruning or blocking GABA_B_ receptor–mediated activation to prevent synaptic loss in epilepsy; by modulating cholinergic and β₂-adrenergic signaling between neurons and macrophages to restore immune balance in inflammatory diseases; and by engaging neuronal PRR pathways for neuroimmune activation in infection or neurodegeneration. As both neurons and innate immune cells transcend their canonical boundaries, they reveal a shared molecular language governing health, disease, and adaptation across the neuroimmune connectome. Ultimately, this convergence signifies not the end of boundaries but the emergence of a new frontier where neurons *act immune* and immunity *acts neural*.
